# BENDR: Using Transformers and a Contrastive Self-Supervised Learning Task to Learn From Massive Amounts of EEG Data

**DOI:** 10.3389/fnhum.2021.653659

**Published:** 2021-06-23

**Authors:** Demetres Kostas, Stéphane Aroca-Ouellette, Frank Rudzicz

**Affiliations:** ^1^Department Computer Science, University of Toronto, Toronto, ON, Canada; ^2^Vector Institute for Artificial Intelligence, Toronto, ON, Canada; ^3^Li Ka Shing Knowledge Institute, St. Michael's Hospital, Toronto, ON, Canada

**Keywords:** brain computer interface, deep learning - artificial neural network, transformers, semi-supervised learning, contrastive learning, convolutional neural network, sequence modeling

## Abstract

Deep neural networks (DNNs) used for brain–computer interface (BCI) classification are commonly expected to learn general features when trained across a variety of contexts, such that these features could be fine-tuned to specific contexts. While some success is found in such an approach, we suggest that this interpretation is limited and an alternative would better leverage the newly (publicly) available massive electroencephalography (EEG) datasets. We consider how to adapt techniques and architectures used for language modeling (LM) that appear capable of ingesting awesome amounts of data toward the development of encephalography modeling with DNNs in the same vein. We specifically adapt an approach effectively used for automatic speech recognition, which similarly (to LMs) uses a self-supervised training objective to learn compressed representations of raw data signals. After adaptation to EEG, we find that a single pre-trained model is capable of modeling completely novel raw EEG sequences recorded with differing hardware, and different subjects performing different tasks. Furthermore, both the internal representations of this model and the entire architecture can be fine-tuned to a *variety* of downstream BCI and EEG classification tasks, outperforming prior work in more *task-specific* (sleep stage classification) self-supervision.

## 1. Introduction

To classify raw electroencephalography (EEG) using deep neural network models (DNNs), these models need to both develop useful features from EEG signals and subsequently classify those features. This frames both the promise and the challenge of using DNNs for supervised EEG classification. On the one hand, it promises to almost entirely circumvent the need for feature engineering, but on the other hand, both feature discovery and classification need to be learned from a *limited*^1^ supply of (relevant) high-dimensional data. A paradigmatic way in which we observe this challenge is with brain–computer interface (BCI) applications^2^ (Lotte et al., 2018; Roy et al., 2019; Kostas and Rudzicz, 2020b). Shallower neural network models have tended to be more effective classifiers than their deeper counterparts in BCI (markedly so when trained independently for each user) (Schirrmeister et al., 2017; Lawhern et al., 2018; Lotte et al., 2018; Roy et al., 2019; Kostas and Rudzicz, 2020b). With these shallower networks, the range of *learnable* features is relatively limited. By design, they employ constrained linear operations, and a limited few include non-linear activations between subsequent layers (Kostas and Rudzicz, 2020b), an otherwise crucial feature of DNN complexity. In prior work, we observed that if some inter-personal variability had been adjusted, the performance of shallower models more quickly saturated to lower performance levels as compared to a deeper network alternative (Kostas and Rudzicz, 2020b), suggesting that more complex raw-BCI-trial features *could* be developed using deeper neural networks when using training data that was more consistent. Understood differently, overcoming the limitations of shallower networks in favor of deeper DNNs that *could* surpass feature engineering approaches likely requires addressing the large variability between different contexts.

A natural framework to understand this problem is transfer learning (TL), which is an area of machine learning that aims to leverage knowledge learned from one context such that it may be useful in a different one. Consider a supervised learning problem, which consists of first, a domain D={X,P(X)}, itself a representation of a feature space X (e.g., the set of all possible raw EEG recordings of a certain length) and the probability *P*(*X*) of observing a particular configuration of features (*x* ∈ *X*, e.g., a particular observation of a raw EEG recording). Second, a task T={Y,f(x)}, a representation of the possible labels for a particular task, and a mapping f:X→Y that maps individual instances to the correct labels. TL means to break down a problem into *source* and *target* problems, with $D_{S}\neq D_{T}$ (and/)or $T_{S}\neq T_{T}$.

Evidence abounds in BCI and EEG generally that differences in domain are a critical challenge. For example, under the sensory motor rhythm (SMR) BCI paradigm, different subjects exhibit extremely different capacities at performing this task, and even different sessions from the same users can exhibit enough variation that classifiers trained in one session are ill-suited to the next (Vidaurre and Blankertz, 2010; Ahn and Jun, 2015; Sannelli et al., 2019). This indicates that (at least for the feature representations being considered) the domain of each person and even session differs. Beyond these inter- and intra-personal variations, different features are relevant for different BCI tasks. Hand-selected features (sets possibly pruned later on) are also typically distinct under different BCI paradigms, as different features better discriminate different tasks^3^ (Lotte et al., 2018), e.g., P300 vs. SMR. Thus, an explicit imposition of difference in domain is imposed between different BCI task paradigms (as their feature spaces are distinct, e.g., $X_{SMR}\neq X_{P300}$), which to us implies that it is fair to expect that this is indicative of strong differences in domain (and of course task) when considering *raw* data. In other words, the very effort of selecting different features for different tasks (rather than only changing classifier) is recognition of a difference in domain. Furthermore, we have found in previous work that the different domains represented by particular individuals seem to be *readily*^4^ identifiable from arbitrary raw sequences of EEG using DNNs (Kostas and Rudzicz, 2020a). In summary, a DNN trained with a certain set of contexts (e.g., subjects), intent on transferable performance to novel contexts (e.g., an unseen subject), is required to develop some universal features and/or classifier for possible novel target domains from the sources it was prepared with. Some have argued that this universality is achievable through the selection of the right DNN, or DNN layers (Cimtay and Ekmekcioglu, 2020; Zhang et al., 2020a), but through a questioning of the apparent ideal approaches to TL in the wider DNN literature (presented in section 1.1), we argue that the development of such universal features requires developing pre-training procedures that transfer from *general tasks* to *specific* ones instead.

The interest in these universal, or invariant features are not however limited to better classification performance, but may be of wider importance. While it may be difficult to determine within a DNN when “features” start and “classifier” begins, in applications such as computer vision there is a clear understanding that nearly all transferrable DNNs have tended to learn “low-level” features in *earlier layers* (e.g., edge-detector-like primitives) (Krizhevsky et al., 2012; Yosinski et al., 2015; Raghu et al., 2019). The promise of some such transferable early layers or operations that are easily extended to any subject, session, or task may open valuable lines of inquiry, or novel explicit (rather than implicitly learned) methods (say if these early layers do or do not correspond to existing methodologies, respectively) of analysis. Importantly, the determination of which “low-level” features DNNs developed in computer vision was revealed through models that *had* transferable performance from general to specific tasks (Yosinski et al., 2015; Raghu et al., 2019).

In this work, we argue that self-supervised sequence learning is such a general task. It would be an effective approach for developing and deploying more complex and universal DNNs in BCI and in potentially wider EEG-based analysis. We present a methodology that can learn from many people, sessions, and tasks using *unlabeled* data; in other words, it samples the more general distribution of EEG data. Thus, we attempt to learn $D_{EEG}$ with self-descriptive features, with the goal that they exhibit little variability across typical context boundaries (invariant between expected domains) like dataset and subjects. More specifically, we investigate techniques inspired by language modeling (LM) that have found recent success in self-supervised end-to-end speech recognition and image recognition in an effort to develop *encephalography models* (EM). We first begin by investigating fully supervised transfer learning (which has been frequently looked to as an EEG/BCI TL solution), finding inconsistency in the extension of computer vision-style pre-training to BCI (and by extension the data domain of EEG). We then evaluate a simple adaptation of previous work in self-supervised speech recognition called wav2vec 2.0 (Baevski et al., 2020) to EEG. With this framework, arbitrary EEG segments are encoded as a sequence of learned vectors we call BErt-inspired Neural Data Representations (or “BENDR”). We ask whether BENDR are transferable to unseen EEG datasets recorded from unseen subjects, different hardware, and different tasks, and how generally suitable BENDR are (both as-is or fine-tuned) to a battery of downstream EEG classification tasks with respect to the same architecture without first being trained with more general EEG data (i.e., “pre-training”).

### 1.1. Pre-training With DNNs

For inspiration on tackling DNN transfer learning in BCI, one can look to other successful approaches, starting with the modern deep learning (DL) “revolution,” which was ushered in on the back of computer vision and image recognition (LeCun et al., 2015; Sejnowski, 2020). The successes of DL in these applications have stemmed from a lineage of massive *labeled* datasets (LeCun et al., 2015), such as the ImageNet dataset (Deng et al., 2009). These datasets were (and are) used to train deep convolutional neural networks, often one of the variants or progeny of ResNet (He et al., 2016) and DenseNet (Huang et al., 2017). Crucially, these are labeled datasets, featuring—especially in the case of ImageNet—an enormous number of unique possible classification labels (or equivalently *targets*, with 1000 being common when using ImageNet^5^, but more are possible^6^). Leveraging labeled data (especially for a singular domain such as a single subject, session and task) of a similar scale in BCI is impractical but, despite this, a sizeable amount of prior work tries to fashion a transfer learning strategy after the successes of ImageNet “pre-training.” These take the form of transferring knowledge from a network *pre-trained* with more data, typically more subjects, to a target domain with less data, typically a single subject (Lin and Jung, 2017; Dose et al., 2018; Schwemmer et al., 2018; Fahimi et al., 2019; Xu et al., 2019; Cimtay and Ekmekcioglu, 2020; Kostas and Rudzicz, 2020b; Zhang et al., 2020a), with some work transferring between entire datasets of the same paradigm, rather than subjects (Ditthapron et al., 2019). On the surface, these embody a *general-to-specific* supervised transfer learning scheme *reminiscent* of ImageNet pre-training where models trained on an ImageNet problem are adapted to a novel (but related) application. However, these particular framings lack the label *diversity* when pre-training with ImageNet. In other words, a narrow set of labels are used to pre-train a model, and these simply overlap with the target context, i.e., $Y_{S}=Y_{T}$. This approach is in fact distinct from the approach taken as inspiration where $Y_{S}\neq Y_{T}$ (or possibly $Y_{S}\subset Y_{T}$). We remain unaware of any work that pre-trains a DNN using a *wide gamut of BCI-relevant targets* in the services of a *more narrow* target set, as would be more analogous to using ImageNet as pre-training toward more specific computer vision tasks^7^. This is noteworthy, as this is what makes ImageNet a *general task*. Evidence suggests that pre-training label diversity is important for effective ImageNet transfer learning (Huh et al., 2016), though an excess could be detrimental (Huh et al., 2016; Ngiam et al., 2018). Furthermore, this general task appears to be responsible for developing the *transferable* early layers (Raghu et al., 2019; Neyshabur et al., 2020) that would seem to embody the desired goal of overcoming “hand-crafted” or developing “invariant” features, and partially appear to be learning data statistics (Neyshabur et al., 2020) [i.e., *P*(*X*); recall this as one aspect of a domain for a supervised learning problem, the other is the feature representation]. More fundamentally, however, this pre-training paradigm has begun to be questioned altogether, with some work finding that it does not necessarily improve downstream performance, where commonly it has been assumed that it should (e.g., in medical images or object localization; though it *speeds up* training considerably) (Ngiam et al., 2018; He et al., 2019; Kornblith et al., 2019; Raghu et al., 2019).

### 1.2. Are There Alternatives?

What has begun to emerge as a potential alternative in computer vision—and markedly so when there is limited labeled downstream data—is self-supervised learning (Chen et al., 2016; van den Oord et al., 2018; Grill et al., 2020; Hénaff, 2020)^8^. These works are inspired by the recent success in natural language processing (NLP) using LMs, which can be used to greatly affect the transfer learning, but also for few-shot and zero-shot learning (Brown et al., 2020; Raffel et al., 2020). These models are understood to work by making a very general model of language and appear even immediately capable of performing tasks they were not explicitly trained to accomplish. We propose that DNN transfer learning in BCI and neuroimaging analysis generally could follow a similar line, with *encephalography models* (EM) in place of LMs. The important question being *how best to construct such an EM so that it learns features that are general enough, while remaining usable for any analysis task?*

To our knowledge, the most similar prior work to this line of inquiry has been the approaches developed for (EEG) self-supervised sleep stage classification (SSC) through contrastive learning (Banville et al., 2019). Contrastive learning is a more particular, yet generally applicable training process that consists of identifying positive representations from a set that also includes incorrect or negative distractor representations (Arora et al., 2019). Banville et al. proposed two potential contrastive learning tasks—a “relative positioning” task and an extension they termed “temporal shuffling” (Banville et al., 2019). Underlying both tasks is the notion that neighboring representations share a label. The representations themselves are a learned mapping (in their case, a convolutional neural network, but ostensibly arbitrary) of raw EEG time-windows to a feature vector. This assumption of similar neighboring labels is fair for SSC, where sleep stages change slowly, and is generally reasonable for continuous problems, where some notion of smoothness can be assumed. Their proposed “relative positioning” task is a binary classification problem distinguishing whether a pair of representations are within a local or positive window τ_*pos*_, or outside a long-range or negative window τ_*neg*_ (when τ_*neg*_ > τ_*pos*_, those falling within τ_*neg*_ but outside τ_*pos*_ are ignored). Their alternative “temporal shuffling” method adds a third window or representation with which to contrast that is within τ_*pos*_ of one (arbitrary) window called the “anchor,” and again learns the representations through a binary classification task. In this case, the classification determines whether the three representations are ordered sequentially, or are out of order. *Downstream* (loose terminology used to mean the step after pre-training when a model is leveraged and evaluated for a particular task), both contrastive learning tasks ultimately improved SSC classification performance over the *same* network trained in a fully supervised manner *from scratch* (with randomly initialized weights rather than those that accomplish the self-supervised task) and their results further agree with the common finding that self-supervision appears distinctly better with limited fine-tuning^9^ data (Brown et al., 2020; Chen et al., 2020). Furthermore, self-supervised pre-training also outperformed an autoencoder-based pretraining, an alternative and historically common pretraining option where a network is pretrained to reconstruct its original input. “Relative positioning” performed better on average (and no statistical significance expressed) when compared to its counterpart, but a linear classification of simple hand-crafted features was still highest performing overall. These results demonstrate the promise of self-supervised learning with DNNs for EEG over a supervised approach, but contextualize them as early in development. This is perhaps best seen by considering the lengths of the time windows (τ_*pos*_ and τ_*neg*_). The shortest windows employed in this particular investigation were 2 min for τ_*pos*_ and τ_*neg*_, which seems prohibitively long for most immediate applications outside of SSC. As it is assumed that representations within τ_*pos*_ are similarly labeled, it may be difficult to expand the use of this technique to time scales closer to that of say, a BCI trial (across any paradigm), which tend to be no more than several seconds at most. In this work, we focus our efforts on adapting a relevant strategy from the wider ML literature that could develop features on smaller time scales effective for BCI trials *as well as* time scales appropriate for SSC.

Returning to a consideration of how one might adapt LM pre-training to EM, the *masked* language model (MLM) is a slight variation on the typical LM that has been essential to the success of recent LMs like BERT (Devlin et al., 2019) and its lineage (Raffel et al., 2020) of similar models. Where a LM estimates the probability of encountering a language token (a word or *sub-word* Aroca-Ouellette and Rudzicz, 2020) given previous (or, in some cases, also subsequent) tokens, a MLM scheme instead learns to *reconstruct* language token(s) given surrounding context (fashioned after the Cloze task). This family of models may deploy a variety of auxiliary tasks (Aroca-Ouellette and Rudzicz, 2020) for transfer learning capabilities, but the task currently at the heart of this family is as follows: given a sequence of *N* tokens *t*_1_, …*t*_*N*_, and a subset of token indexes *I*_*m*_, for each token index *i* ∈ *I*_*m*_, tokens are masked with some mask *M* so that:

(1) $$q_{i}=\left\{ \begin{array}{l} M;i\in I_{m}\\ t_{i};\text{otherwise} \end{array}\right.\;,\forall i\in N$$

A transformer encoder (Vaswani et al., 2017; Devlin et al., 2019) then reconstructs the original sequence of tokens from the *masked* sequence [*t*_*i*_ and *q*_*i*_, ∀*i* ∈ *N*, respectively, in Equation (1)]. *M* could be a single learned token (Baevski et al., 2020), or in the case of BERT: 80% of the time a fixed [MASK] token, 10% a random token or 10% the original token (with 15% of tokens masked within each sequence) (Devlin et al., 2019).

Could an EM be developed in this vein, using say, individual samples rather than tokens (i.e., could a direct application of the above be done with raw EEG)? Unfortunately, the highly correlated nature of neighboring samples in EEG (or most other continuous data for that matter) is not conducive to this approach. The likely result would be that, instead of an EM, a method for interpolation would be learned, the model would simply learn how to average neighboring samples, as has been argued in similar work in self-supervised learning with speech (Jiang et al., 2020). In other words, the smoothness of these data would make it hard to produce general features simply through recovering missing individual samples. Masking a contiguous span of tokens instead, which is beneficial in NLP (Joshi et al., 2020; Raffel et al., 2020), could avoid simply learning to interpolate missing samples, but the *reconstruction* of time-series data is difficult, due to the challenge (among other things) of capturing the degree of error in time (within contiguous sequences) (Rivest and Kohar, 2020). The losses used for such reconstruction, commonly mean squared error (or mean absolute error), erroneously assume independence in the error between elements in the series, causing inappropriate error signals when (among other things) simply shifting a reconstruction in time (Rivest and Kohar, 2020).

Contrastive predictive coding (CPC), is a particular contrastive learning approach that is intended for sequence learning. With CPC, the correct *learned representation* for a particular sequence offset is predicted relative to distractor representations, typically those of other positions in the same sequence (van den Oord et al., 2018). What is notable about this is that it is not as susceptible to degeneration into interpolation, nor is it similarly affected by the issues of time-series reconstruction (van den Oord et al., 2018). This task enables learning both a good feature representation *and* an understanding of the sequence of data by modeling the progression of the representations, learned with a single loss function. Indeed, the RP and TS tasks discussed above for SSC can be understood as special cases of the more general CPC, though performance appears largely similar when comparing all three (Banville et al., 2020).

Prior work in self-supervised *speech recognition* has begun to synthesize parts of CPC and MLM to produce methodologies for self-learning with raw waveforms (van den Oord et al., 2018; Baevski and Mohamed, 2020; Baevski et al., 2020; Chung et al., 2020; Jiang et al., 2020). In our work, we adapt one of these approaches called wav2vec 2.0 (Baevski et al., 2020) (its particular formulation is detailed in section 2.4.1) to EEG. We consider how efficient the approach is at developing representations (BENDR), and how general these and the accompanying sequence model are across multiple task paradigms/datasets (not seen during pre-training) and across the subjects that constitute them. Since interestingly, both the representations alone (Chen et al., 2020), and the addition of the sequence model (Baevski et al., 2020) have proven potentially useful for supervised fine-tuning *after* pre-training, we then characterize a variety of “fine-tuning” approaches “downstream.” In other words, finally, we compare which aspect of our overall scheme is best leveraged and how toward classifying a variety of publicly available EEG classification task datasets.

## 2. Materials and Methods

All experiments are implemented using the *deep neural networks for neurophyisiology* (DN3) library^10^. The source code and pre-trained BENDR models can be found at https://github.com/SPOClab-ca/BENDR.

### 2.1. Datasets

#### 2.1.1. Pre-training

We intend to learn our proposed general task across a large number of typically confounding domains, which means the ideal pre-training dataset for our purposes would feature many subjects, each recorded over many sessions. These sessions would also ideally be distributed across large time scales and consist of a variety of performed tasks. In other words, the pre-training dataset should consist of a representative sample of EEG data. This also means that these data should include multiple different recording hardware and configurations. The closest publicly accessible dataset, to our current knowledge, was the Temple University Hospital EEG Corpus (TUEG) (Obeid and Picone, 2016). It consists of clinical recordings using a mostly conventional recording configuration (monopolar electrodes in a 10–20 configuration) of over 10,000 people, some with recording sessions separated by as much as 8 months apart. The subjects were 51% female, and ages range from under 1 years old to over 90 (Obeid and Picone, 2016). We focused specifically on versions 1.1 and 1.2 of this dataset which amounted to approximately 1.5 TB of European-data-format (EDF) EEG recordings *before* preprocessing.

#### 2.1.2. Downstream

To investigate the practical utility of the learned representations, we compiled a non-exhaustive battery of publicly accessible EEG data classification tasks—or *downstream tasks*—summarized in Table 1. Most of these were BCI task datasets, which could readily be compared to previous work with DNNs trained without any additional unlabeled data (Lawhern et al., 2018; Kostas and Rudzicz, 2020b). We also included one of the SSC tasks used by Banville et al. (2019) in their work on sleep stage self-supervision described above, for comparison. This particular dataset afforded some further insight into generality, as BCI data are typically classified in the context of particular trials or events, and SSC is a more continuous problem, requiring that large spans of time are labeled with the particular sleep stage a subject is undergoing. These segments are distinctly longer than the BCI trials we considered in the remaining battery (an order of magnitude difference in our case when compared to the largest BCI task sequence length), and are distinctly closer in length to the way the pre-training task is formulated (see section 2.4.1). We specifically segmented these sequences into periods of 30 s to be classified into 5 sleep stages as in prior work (Banville et al., 2019; Mousavi et al., 2019). Another potentially notable difference with the SSC dataset was the scale of available labels, which seems to have enabled prior work to consider deeper and more complex models (Mousavi et al., 2019).

**Table 1:** Summary of downstream dataset battery and number of cross-validation folds used.

**Dataset**	**Paradigm**	**sfreq. H*z***	**# Ch**.	**Subjects**	**Targets**	**Folds**
MMI Goldberger et al. (2000); Schalk et al. (2004)	MI (L/R)	160	64	105	2	5
BCIC Tangermann et al. (2012)	MI (L/R/F/T)	250	22	9	4	9
ERN Margaux et al. (2012)	Error related negativity	200	56	26 (10)	2	4
P300 Goldberger et al. (2000); Citi et al. (2010, 2014)	Donchin speller	2,048	64	9	2	9
SSC Goldberger et al. (2000); Kemp et al. (2000, 2018)	Sleep Staging	100	2	83	5	10

*Cross-validation splits were in a leave-multiple-subjects-out configuration if Folds < Subjects, or leave-one-subject-out if Folds = Subjects (as in prior work Kostas and Rudzicz, 2020b). The ERN dataset was featured in an online competition^11^ which featured 10 held-out test subjects (not used during training), which we used as a test dataset for all four validation splits of this dataset*.

### 2.2. Preprocessing

The focus of the preprocessing stage was to create a maximally consistent representation of EEG sequences across datasets (which implied differences in hardware), so that a pre-trained network was well suited to the downstream tasks. More or less, this amounted to modifying downstream datasets to match the configuration of the pre-training dataset. The first aspect of this was to remove spurious differences in channel amplitude. Each sequence gathered for training was linearly scaled and shifted (a weight and offset for each sequence adjusts every sample in the sequence) so that the maximum and minimum values within each sequence equal 1 and −1, respectively. To account for the lost relative (to the entire dataset) amplitude information, a single channel was added with the constant value $\frac{max\left(s_{i}\right)-min\left(s_{i}\right)}{max\left(S_{ds}\right)-min\left(S_{ds}\right)}$, where *S*_*ds*_ is the set of all samples in the dataset and *s*_*i*_ ⊂ *S*_*ds*_ is a particular sub-sequence (i.e., trial). We additionally addressed the differences in sampling frequency and electrode sets of the different dataset. Our solutions to these problems were similarly minimalist and were achieved using standard features in DN3 (Kostas and Rudzicz, 2020a). Specifically, we over- or undersampled (by whole multiples, for lower and higher sampling frequencies, respectfully) to get nearest to the target sampling frequency of 256 H*z*. Then, nearest-neighbor interpolation was used to obtain the precise frequency (as was done in prior work Kostas and Rudzicz, 2020a). Additionally, the P300 dataset was low-pass filtered below 120 H*z* to avoid aliasing due to its higher sampling rate (and associated higher original low-pass filter). Furthermore, the SSC dataset featured two bi-polar electrodes: FPz-Cz and Pz-Oz, which were simply mapped to FPz and Pz, respectively. The TUEG dataset itself featured some higher sampling rate signals; we included those with low-pass filters that did not violate the Nyquist criterion (and subsequently re-sampled them as above), and ignored the rest.

A reduced subset of the Deep1010 channel mapping from DN3 (Kostas and Rudzicz, 2020a) was used throughout. This ensured that particular channels were mapped to a consistent index for each loaded trial. The original mapping was designed to be more inclusive, and thus assumed up to 77 possible EEG electrodes. In the interest of minimizing unnecessary electrodes for an already high-dimensional problem, we focused on the 19 EEG channels of the *unambiguously illustrated 10/20* channel set (UI 10/20) (Jurcak et al., 2007), as the TUEG dataset recordings were done using a roughly 10/20 channel scheme. We simply ignored reference electrodes, electro-oculograms, and any other auxiliary channels. When also accounting for the additional relative amplitude channel described above, every sequence from every dataset used 20 channels. All surplus channels were ignored, and missing channels set to 0.

During pre-training, we extracted sequences of 60 s (every 60 s) from each usable sequence, which amounted to 15, 360 samples per subsequence. We observed in early testing that there was better performance with larger sequences (see Figure 4 for more). As can be seen in Table 2, the downstream datasets all classified sequence lengths shorter than this, but the architecture we employed (see section 2.3) was ostensibly agnostic to sequence length (see section 4 for caveats).

**Table 2:** Performances of downstream datasets.

**Dataset**	**Start (s)**	**Length (s)**	**Metric**	**Best**	**Model config**.
MMI	0	6	BAC	86.7	Linear (2.)
BCIC	–2	6	Accuracy	42.6	Linear (2.)
ERN	–0.7	2	AUROC	0.65	Linear (2.)
SSC	0	30	BAC	0.72	Linear (2.)
P300	–0.7	2	AUROC	0.72	BENDR (1.)

*Start and length refer to length of trials and start with respect to event markers in seconds. Best performance specifies average performance across all subjects (and therefore folds) for best performing model configuration. BAC: class balanced accuracy; AUROC: area under the receiver operating characteristic curve. Model configurations are numbered in accordance with the list presented in section 2.4.2*.

### 2.3. Model Architecture

**Figure 1:**
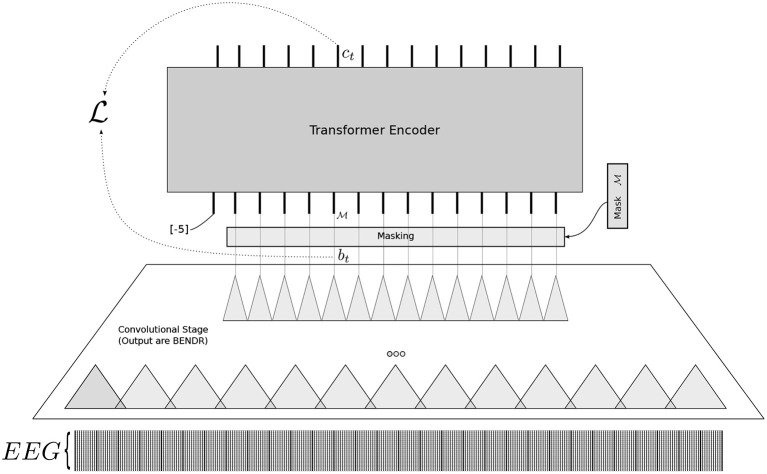
The overall architecture used to construct BENDR. Loss L is calculated for a masked BErt-inspired Neural Data Representations (BENDR) *b*_*t*_ (after masking, it is replaced by the learned mask M), itself produced from the original raw EEG (bottom) via a progression of convolution stages. The transformer encoder attempts to produce *c*_*t*_ to be more similar to *b*_*t*_ (despite that it is masked) than it is to a random sampling of over BENDR.

The model architecture displayed in Figure 1 closely follows that of wav2vec 2.0 (Baevski et al., 2020) and is composed of two stages. A first stage takes raw data and dramatically downsamples it to a new sequence of vectors using a stack of short-receptive field 1D convolutions. The product of this stage is what we call BENDR (specifically in our case, when trained with EEG). A second stage uses a transformer *encoder* (Vaswani et al., 2017) (layered, multi-head self-attention) to map BENDR to some new sequence that embodies the target task.

Raw data are downsampled through the stride (number of skipped samples) of each convolution block in the first stage (rather than pooling, which would require greater memory requirements). Each of our convolution blocks composed of the sequence: 1D convolution, GroupNorm (Wu and He, 2020), and GELU activation (Hendrycks and Gimpel, 2016). Our own encoder features six sequential blocks, each with receptive fields of 2, except for the first block, which has 3. Strides match the length of the receptive field for each block. Thus, the *effective sampling frequency* of BENDR is 96 times smaller (≈ 2.67 H*z*) than the original sampling frequency (256 H*z*). Each block consists of 512 filters, meaning each resulting vector has a length of 512.

The transformer follows the standard implementation of Vaswani et al. (2017), but with internal batch normalization layers removed and with an accompanying weight initialization scheme known as T-Fixup (Huang et al., 2020). Our particular transformer architecture uses 8 layers, with 8 heads, model dimension of 1536 and an internal feed-forward dimension of 3076. As with wav2vec 2.0, we use GELU activations (Hendrycks and Gimpel, 2016) in the transformer, and additionally include LayerDrop (Fan et al., 2019) and Dropout at probabilities 0.01 and 0.15, respectively, during pre-training but neither during fine-tuning. We represent position using an additive (grouped) convolution layer (Mohamed et al., 2019; Baevski et al., 2020) with a receptive field of 25 and 16 groups before the input to the transformer. This allows the entire architecture to be sequence-length independent, although it may come at the expense of not properly understanding position for short sequences.

Originally, the downstream target of the wav2vec 2.0 process was a speech recognition *sequence* (it was fine-tuned on a sequence of characters or phonemes) (Baevski et al., 2020). Instead, here the entire sequence is classified. To do this using a transformer, we adopt the common practice (Devlin et al., 2019) of feeding a fixed token (*a.k.a*. [CLS] in the case of BERT or, in our case, a vector filled with an arbitrary value distinct from the input signal range, in this case: −5) as the first sequence input (prepended to BENDR). The transformer output of this initial position was not modified during pre-training, and only used for downstream tasks.

The most fundamental differences in our work as compared to that of the speech-specific architecture that inspired it are as follows: (1) we do not quantize BENDR for creating pre-training *targets*, and (2) we have *many* incoming channels. In wav2vec 2.0, a *single* channel of raw audio was used. While a good deal of evidence (Schirrmeister et al., 2017; Chambon et al., 2018; Lawhern et al., 2018; Lotte et al., 2018; Kostas et al., 2019; Kostas and Rudzicz, 2020b) supports the advantage of temporally focused stages (no EEG channel mixing) separate from a stage (or more) that integrates channels, we elected to preserve the 1D convolutions of the original work to minimize any additional confound and to reduce complexity (compute and memory utilization ∝ *N*_*filters*_ with 2D rather than $\propto \frac{N_{filters}}{N_{EEG}}$ for 1D convolutions). This seemed fair, as there is also evidence that 1D convolutions are effective feature extractors for EEG, particularly with large amounts of data (Gemein et al., 2020; Kostas and Rudzicz, 2020a). Notably, wav2vec 2.0 downsampled raw audio signals by a much larger factor (320) than our own scheme, but speech information is localized at much higher frequencies than encephalographic data are expected to be. The new effective sampling rate of BENDR is ≈ 2.67 H*z*, or a feature-window (no overlap) of ≈ 375*ms*. We selected this downsampling factor as it remained stable (i.e., it did not degenerate to an infinite loss, or simply memorize everything immediately) during training.

### 2.4. Training and Evaluation

We used the Adam (Kingma and Ba, 2015) optimizer throughout training (during pre-training and fine-tuning with downstream data), with weight decay set to 0.01. We additionally used a cosine learning rate decay with linear warm-up for 5 and 10% of total training steps (batches) for pre-training and fine-tuning, respectively. The peak learning rate itself varied by dataset; this and other variable hyperparameters are further documented in Appendix A.

#### 2.4.1. Pre-training

The pre-training procedure largely follows wav2vec 2.0 but we make some notable hyperparameter changes documented below. The procedure itself is as follows: first, the convolutional stage of the overall architecture develops a sequence of representations (in our case BENDR) that summarizes the original input. An input token is prepended to this sequence (a BENDR-lengthed vector filled with −5), and contiguous spans of the remaining sequence are masked. This modified sequence is provided as input to the transformer stage, which is expected to develop outputs that are *most similar* to the *un-masked* input at a position *t*. Specifically, we use the self-supervised loss function for a masked token localized at *t*:

(2) $$ℒ=-log\frac{exp(cossim(c_{t},b_{t}))/\kappa }{\sum_{b_{i}\in B_{D}}exp(cossim(c_{t},b_{i}))/\kappa }$$

where *c*_*t*_ is the output of the transformer at position *t*, *b*_*i*_ is the (original/un-masked) BENDR vector at some offset *i*, and *B*_*D*_ is a set of 20 uniformly selected distractors/negatives from the same sequence, plus *b*_*t*_. We use the cosine similarity *cossim*(*x, y*) = *x*^*T*^*y*/(|*x*||*y*|) function to determine how similar vectors are, and the sensitivity of this is adjusted by a temperature factor *κ*, set to 0.1. This loss is expected to operate by adjusting the output of the transformer at position *t* to be *most similar to the encoded representation at* *t, despite that this input to the transformer is masked*. This means the transformer must learn a general enough model of *BENDR* (not EEG *per se*) such that the entire sequence of BENDR can characterize position *t* well. We also add the mean squared activation of the BENDR to the loss to keep the activations from growing too large, as was similarly done previously (Baevski et al., 2020), but we set the weight of this additional term to 1 (rather than 10).

Contiguous sequences of 10 BENDR are masked before input to the transformer with probability *p*_*mask*_ = 0.065, such that, for each sample, the likelihood of being the *beginning* of a contiguous section was *p*_*mask*_, and overlap is allowed. We learn a single mask vector during pre-training of the same length as each BENDR vector, and use this as the transformer input to masked positions; masking is done by replacing a masked BENDR with a learned vector. The number of negatives/distractors was set to 20 and uniformly sampled from the *same* sequence as the masked vector, i.e., negatives do not cross trials or sequences.

To evaluate how generalizable the sequence model and vectors were to unseen data *after* pre-training, we evaluated the contrastive task, expressed as the transformer accuracy in constructing *c*_*t*_ to be most similar to *b*_*t*_ rather than the distractors/negatives, with respect to unseen data (in this case the downstream datasets). Note here that no further training or any evaluation with respect to downstream task labels was performed. This was done to evaluate the variability of the representations after pre-training. During this evaluation step, we masked half the amount expected during training, but did so such that masked spans were evenly spaced through the sequence (so that there were no overlapping sequences, and sufficient context was available). That is, for a sequence length of *N*_*S*_, we masked 0.5 × *N*_*S*_ × *p*_*mask*_ = *N*_*m*_ contiguous sequences (of 10), and spaced them every $\left\lfloor \frac{N_{S}}{N_{m}}\right\rfloor$ steps (starting at the first sample). *N*_*S*_ first remained at 15, 360 (60 s as in training, no overlap between subsequent sequence representations) for all datasets except P300, where sessions were too short and instead 5, 120 (20s) was used. We then evaluated the change in performance across the downstream datasets, excluding P300, as *N*_*S*_ varied from 20 to 60 s.

#### 2.4.2. Downstream Fine-Tuning

Ultimately, our aims for subject-, session-, and dataset-generalizable representations were not simply to accurately select for the correct input (what was evaluated of the pre-training BENDR and sequence model), but with the intent that these representations (BENDR)—and potentially the sequence model itself—could be effectively transferred to specific and arbitrary tasks. We considered six different variations of TL across the battery of downstream EEG classification tasks (classification tasks listed in Table 1):

1. Add a new linear layer with softmax activation (classification layer) to the first (recall this position was pre-pended with an *input* value of −5 to the BENDR) output token of the transformer. Then, fine-tune the entire model (continue training the pre-trained model and start training the new layer) to classify the downstream targets using the output of this layer (ignoring the remaining sequence outputs) (shown in Figure 2.1).
2. Ignore the pre-trained transformer entirely, and use only the pre-trained convolutional stage (i.e., only use the BENDR). Create a consistent-length representation by dividing the BENDR into four contiguous sub-sequences, average each sub-sequence and concatenate them^12^. Add a new linear layer with softmax activation to classify the downstream targets with respect to this concatenated vector of averaged BENDRs (shown in Figure 2.2).
3. The same as Figure 2.1, but perform no pre-training; start with a randomly initialized DNN, as shown in Figure 2.3.
4. The same as Figure 2.1, but keep the BENDR (convolutional stage) fixed and continue training the transformer (and start training the new classification layer) to classify downstream targets, as shown in Figure 2.4.
5. The same as Figure 2.2, but perform no pre-training; start with randomly initialized convolution stage, as shown in Figure 2.5.
6. The same as Figure 2.2, but keep the first stage weights fixed and train only the new classification layer, as shown in Figure 2.6.

**Figure 2:**
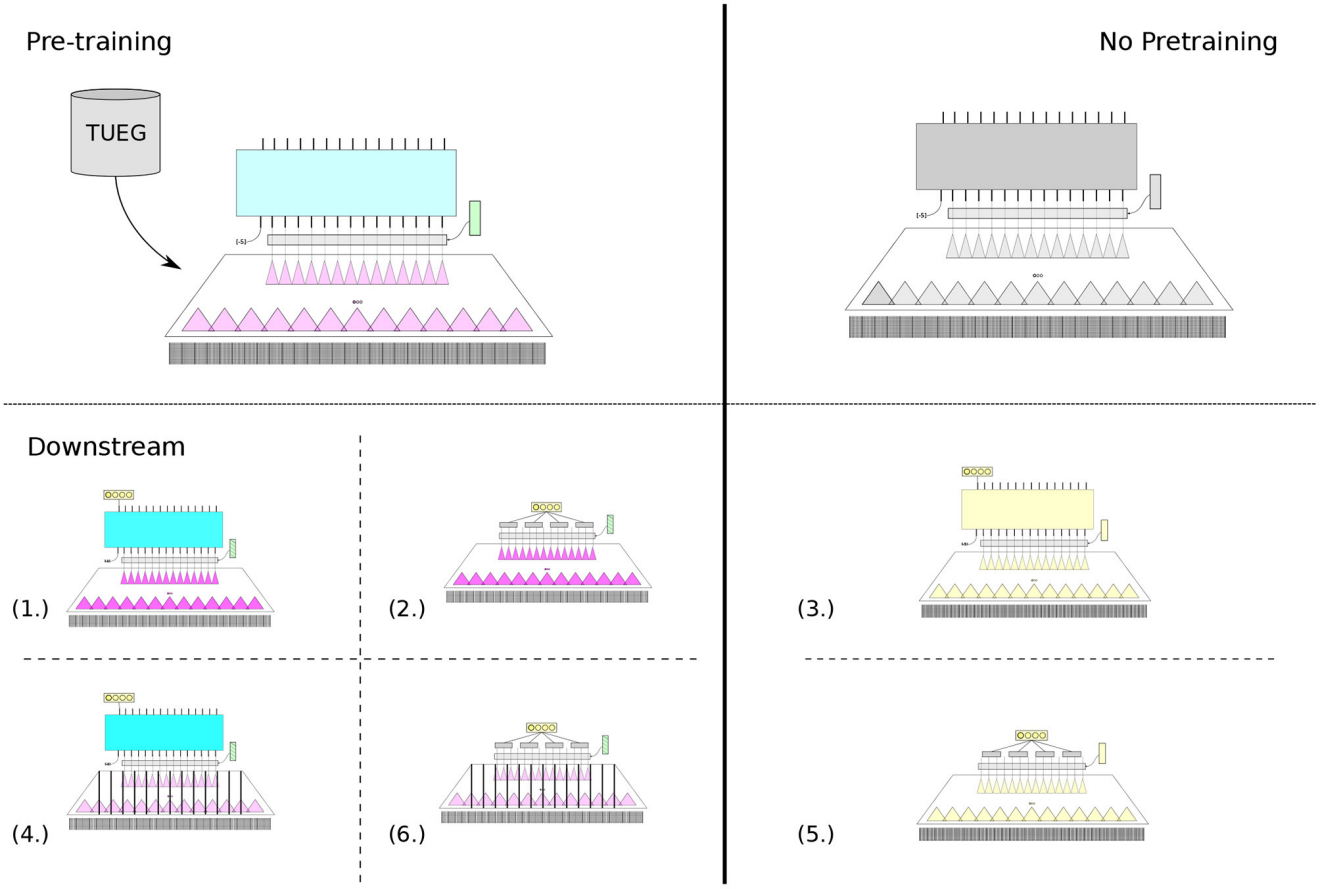
Six different permutations of the model architecture were trained with conventional fully supervised training (in a leave-one/multi-subject-out fashion, see Table 1) for each downstream task. Indicated here is the portion of the overall architectures used (see Figure 1), and how pre-training model weights were leveraged for a four-way classification task (rectangle with four circles in it). Four tasks (left half) leveraged model weights that were first developed through pre-training. All *yellow* modules here indicate randomly initialized weights. Color that progresses in intensity (from pre-training to downstream) indicates further training, while added bars indicate weights that were kept unchanged during that training stage.

Figure 2 provides some illustration of each variation, where the respective indexed subfigures correspond to the list numbers above. These were considered so that we could speak to the effect each stage had on downstream performance, at least to some degree. We were interested in (1) determining whether the new sequence representation (BENDR) contained valuable features *as-is* (as they appear to be for speech Baevski et al., 2020) or if they required specific adaptation, and (2) whether the sequence model learned characteristics of the BENDR that were informative to the classification task. Finally, ignoring pre-training all-together, of course, was to examine how effective the network would be at learning the task otherwise, without the general pre-training task.

At this stage, we also included the sequence regularization proposed by wav2vec 2.0 (Baevski et al., 2020), although we adjusted it for our more varied trial lengths. That is, in all 6 fine-tuning configurations, contiguous sections of 10% of the entire BENDR of a trial were masked with the mask token learned during pre-training (not changed after pre-training) at a probability of 0.01. In other words, this was the likelihood of a sample being the beginning of a contiguous masked section, as in pre-training. Additionally across the BENDR (throughout each vector in the sequence), a similar procedure dropped features to 0, where contiguous sections of 10% of the channels (51) were dropped with a probability of 0.005.

The P300, ERN, and SSC datasets all had imbalanced class distributions; during training, we adjusted for these imbalances by *undersampling* points uniformly of the more frequent classes with replacement so that the number of samples drawn—per epoch—of each class was equal to the number of examples of the least frequent target class. As the test conditions then were imbalanced, test performance was evaluated using metrics that accounted for this, and followed previous work (Baevski et al., 2020; Kostas and Rudzicz, 2020b). Metrics are specified by dataset in Table 2.

## 3. Results

### 3.1. Pre-training Generalization

**Figure 3:**
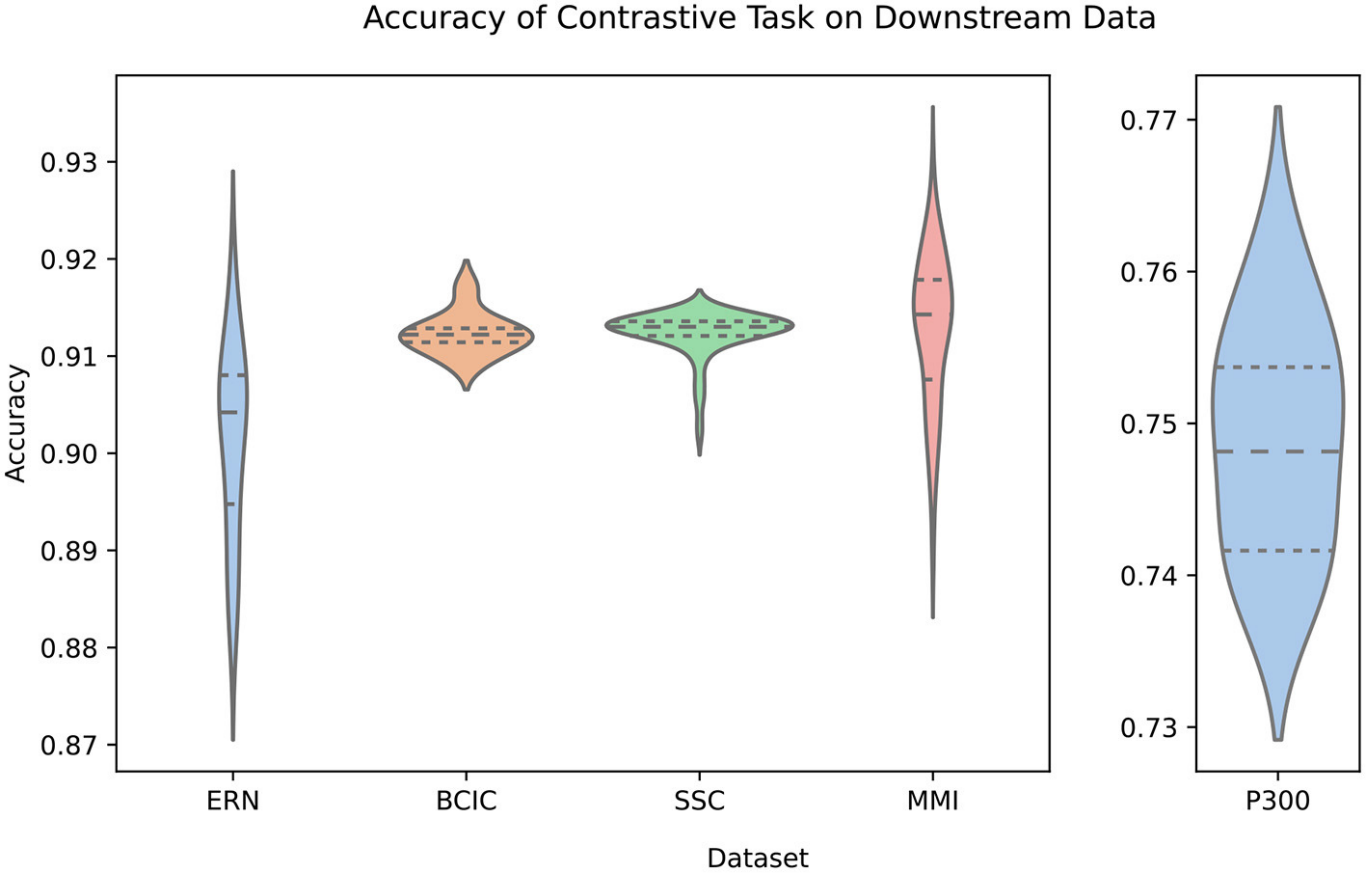
Violin plot (inner lines for quartile divisions) of test subject-wise accuracy for each downstream dataset. Specifically, accuracy of the sequence model (transformer stage) at creating a representation that is closest to the correct representation at masked sequence positions. The P300 dataset is distinctly lower performing (note the adjusted *Y*-axis) than the remaining datasets, though this was likely due to its shorter evaluation context (see Figure 4). Nonetheless, there is minimal test subject-wise variation, particularly when compared to classifier performance generally.

**Figure 4:**
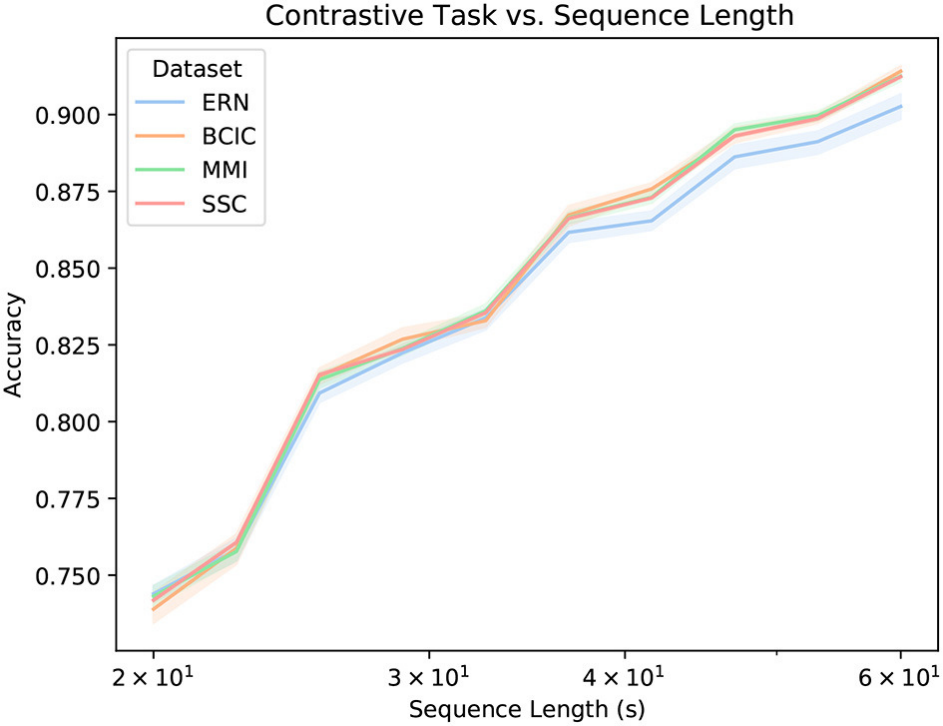
Contrastive accuracy vs. evaluation length in seconds (*x*-axis logarithmic). Performance is distinctly similar for all datasets, rising for longer sequences. We suggest that this implies that samples that are further apart are easier to distinguish between than neighboring samples. Thus, while BErt-inspired Neural Data Representations (BENDR) encode local signal characteristics well, there is redundancy.

**Figure 5:**
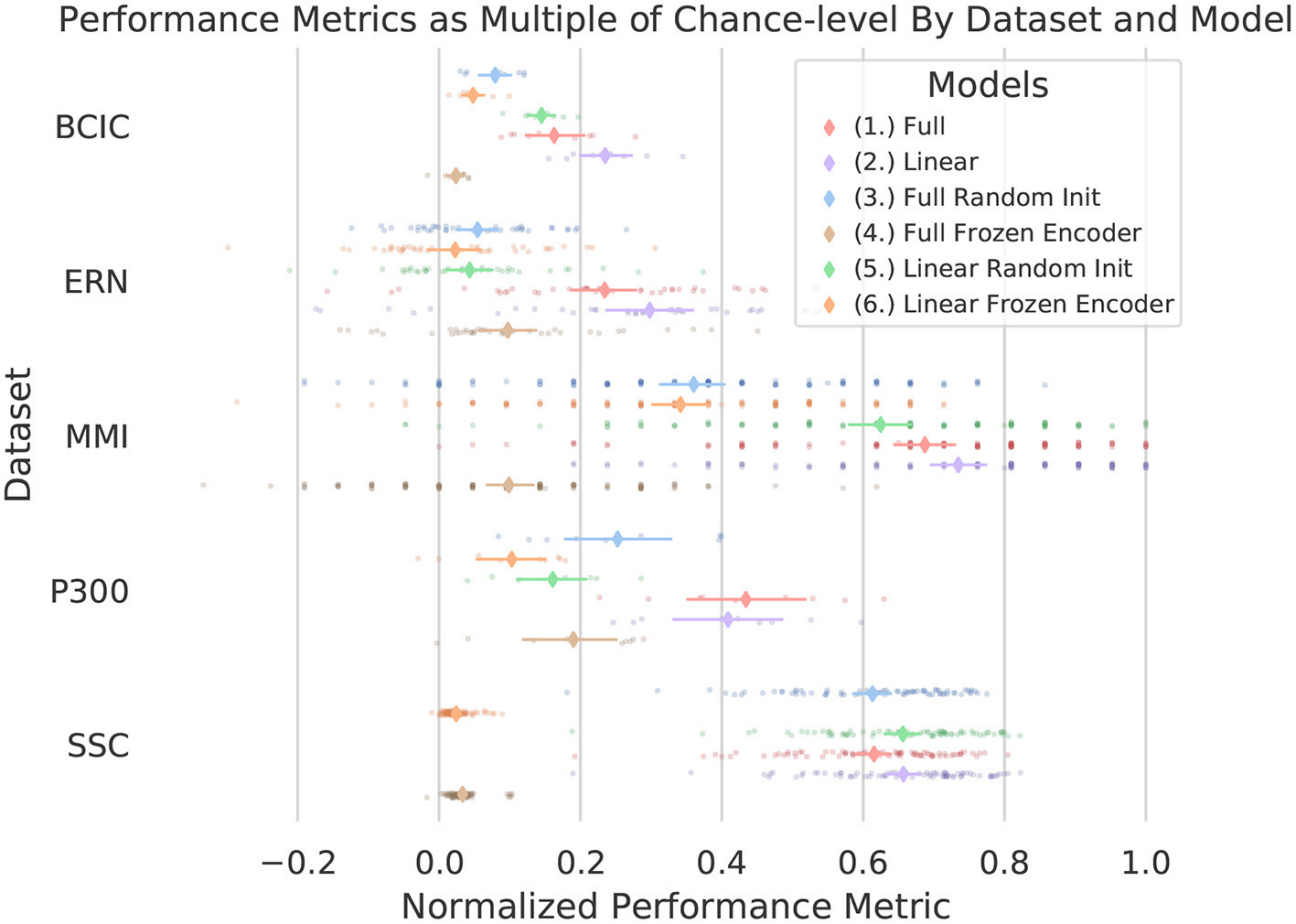
Performance of all downstream datasets for each of the six model configurations considered. Metrics vary by dataset, see Table 2. Metrics were normalized to range from chance (0) to perfect (1). Individual translucent points are performances of single subjects (within each test fold), solid diamonds indicate mean performance across all subjects/folds, with surrounding bars showing 0.95 confidence intervals using *n* = 1000 bootstrap sampling. The discretized pattern of the MMI dataset is due to the limited trials *per subject*, which resulted in limited distribution of performance levels. Notably here, (1) or (2) was consistently among the best performing, yet both remained within the confidence levels of each other and aside from a few cases with the ERN dataset did not result in subject performances that were worse than chance. (3) and especially (4) and (6) often stayed marginally above chance, indicating that the pre-trained features were not sufficient without further training. The randomly initialized average-pooled BENDR with linear classifier (5) also performed well, though less consistently, suggesting pre-training was needed for *consistent* performance. Model configurations are numbered in accordance with the list presented in section 2.4.2.

Figure 3 shows how accurate the transformer stage is at producing an appropriately similar BENDR. There are two key observations in this figure, the first is that there is little variability across the first four datasets, and within each of the five datasets. The latter point implies that this accuracy is not radically variable across different subjects (though, when fine-tuning for classification, this variability returns; see Figure 5). This could be because (a) the transformer adequately learns a general model of how BENDR sequences of novel persons and equipment progressed; (b) the BENDR themselves are invariant to different people, hardware, and tasks; (c) some combination of the last two possibilities; or (d) the problem is being solved via some non-signal characteristics. We return to this question shortly. The second observation was already alluded: the P300 dataset distinctly under-performs the other downstream datasets. However, this coincided with the shortest evaluation sequence. Looking at Figure 4, we see that all five datasets have consistently similar performance when evaluated with 20 s of data, so the dip in P300 performance of Figure 3 seems less remarkable. Taken together, Figures 3, 4 clearly indicate that a longer evaluation context makes the contrastive task easier. This suggests that the contrastive task is, in fact, solved by learning signal-relevant features, rather than some more crude solution like interpolation, or by simply creating a sequence of recognizable position representations (both of which have no reason to exhibit this dependence on sequence length). We believe that the most likely explanation for the *rise* in performance with more context is that local representations are more difficult distractors, implying that the new effective sampling rate remains too high (and there is still redundant information encoded in local BENDR). Notwithstanding, there is a strong uniformity of performance across datasets and subjects (in both Figures 3, 4), meaning this scheme develops features (whether through the transformer itself, or the BENDR) that generalize to novel subjects, hardware, and tasks, though their applicability to downstream contexts remains to be seen.

### 3.2. Downstream Fine-Tuning

Figure 5 and Table 2 present a picture of how effectively BENDR could be adapted to specific tasks. Overall, the fine-tuned linear classification (the downstream configuration in Figure 2.2) that bypassed the transformer entirely after pre-training was highest performing four out of five times, although using the transformer for classification (Figure 2.1) performed consistently similarly (confidence intervals always overlapped), and surpassed the bypassed transformer (Figure 2.2) with the P300 dataset (and was highest performing for this dataset). Deploying the full network (initial stage and transformer) *without pre-training* was generally ineffective, though this was not the case with the SSC dataset, which may have been due to the larger amount of data available for fully supervised learning. In fact, for both the full and linear model architectures trained with the SSC data, fine-tuning the pre-trained model is mostly on par with the fully supervised counterpart. Considering our results with the SSC data relative to those of Banville et al. (2019) proposed contrastive learning for sleep staging (described in section 1.1), their reported results show that the fine-tuned variants of our own model (1 and 2) achieved a higher mean balanced accuracy relative to their two proposed schemes. Taken in concert with our own approach's wider applicability and more fine-grained temporal feature development, we believe this demonstrates that ours is a promising alternative. Interestingly, with and without pre-training (Figure 2.2, 2.5) achieved similar performance to Banville et al.'s fully supervised results (where our configurations and their architecture employ similar 1D convolution-based schemes), which is notable as with this dataset, both their “temporal-shuffling” and “relative-positioning” tasks under-performed this full supervision performance level (though we cannot speak to statistical significance of this comparison).

Our fine-tuned approaches similarly appear reasonably competitive with prior work on the MMI dataset (Dose et al., 2018; Kostas and Rudzicz, 2020b), particularly when considering that only 19 channels (rather than the full set of 64) were being used. Outside of the MMI and SSC dataset, remaining results are not competitive with more targeted solutions (Kostas and Rudzicz, 2020b). Whenever pre-training was not used, despite heavy regularization (and the very low learning rates) the randomly initialized parameters were consistently prone to overfitting, all the more so with the full model architecture. Conversely, the pre-trained networks were slow to fit to the downstream training data (under the exact same training scheme for fine-tuning). Despite that these results were not necessarily state of the art, this single pre-training scheme nonetheless shows a breadth of transferability that is apparently unique, and aside from the SSC dataset, consistently here outperforms the fully supervised counterparts.

## 4. Discussion

We are unaware of any prior work assessing transformer-based (Vaswani et al., 2017) DNNs with EEG data (raw or otherwise). This is perhaps consistent with the ineffectiveness we observed with the *randomly initialized* full architecture (Figure 2.3) and could imply that effective use of this powerful emerging architecture *requires* pre-training (or at least enough data, given the better looking SSC performance). This may be due to the large number of parameters that these models require, making training difficult without sufficient hardware resources. The total number of parameters trained in configuration (1) is over one billion parameters. Future work should continue to evaluate this architecture, particularly as it appears to be more widely applicable than the NLP applications it was originally proposed for (Baevski et al., 2020; Dosovitskiy et al., 2020).

We believe that our approach can be improved through adjusting the neural network architecture and pre-training configuration such that it becomes more data-domain (EEG) appropriate. Future work will prioritize effective integration of spatial information, likely by better isolating temporal and spatial operations. Evaluation using large downstream datasets that *also* feature many channels, such as the *Montreal Archive of Sleep Studies* (MASS)^13^ will be considered. Though available for public access at the time of writing, these data were unavailable while experiments were prepared and conducted. Prior work shows that DNN approaches effective for EEG leverage spatial information (Chambon et al., 2018), and it is presently unclear to what degree this is the case with BENDR. In terms of data-appropriate temporal modeling, which we have considered with relatively more zeal in this work, recall that Figure 4 presents the possibility that local representations may be retaining redundant information, further improvements therefore may be found in better compressing the temporal resolution of BENDR. Future work will consider larger downsampling factors in the initial stage, along with longer sequences, balancing the more difficult problem of summarizing more data (in effect, further data *compression*), with the apparent increased effectiveness of the contrastive task (as observed in Figure 4) on longer sequences. A small but potentially fruitful avenue for further improvement includes reconsidering the additive convolutional layer as a substitute for explicit position encodings, which are in fact more common (Vaswani et al., 2017; Devlin et al., 2019; Raffel et al., 2020). Recall that this was originally for two reasons: wav2vec 2.0 did the same, and we felt it best to limit excessive changes to the architecture on a first iteration, and also because it seamlessly supported flexible input lengths. This latter point comes however, with a trade-off: our particular position encoder had a receptive field of 25 (stride of 1), which means a little over 9 s of input. While it seems that convolutional position encodings offer better performance (Mohamed et al., 2019), this input width exceeded the *entire* length of all but the sleep classification task (the length we chose was optimized for pre-training behavior).

After considering these possible avenues for improving BENDR, we still do not fully discount the validity of some of the transfer learning paths we appear to exclude above in our introduction. We will reconsider these paths in future work. Particularly, given the success we had in crossing boundaries of hardware in this work, and in prior work (Kostas and Rudzicz, 2020a), it may be possible to construct an *aggregate* dataset featuring a variety of EEG classification tasks toward better ImageNet-like pre-training. The construction of a more coherent label set that crosses several BCI paradigms would no doubt be a significant effort (e.g., problems may include: is a rest period before one task paradigm the same as rest before another? What about wakeful periods in sleep?). This would no doubt be imbalanced; the labels would be distributed in a long-tailed or Zipfian distribution that would likely require well thought-out adjustment (Cao et al., 2019; Tang et al., 2020).^13^ Furthermore, the value of ImageNet pre-training *seems to be* localized to very early layers and the internalization of domain-relevant data statistics (Raghu et al., 2019; Neyshabur et al., 2020). Future work could look into which of these may be leveraged with a new aggregate (multiple subjects *and* tasks) pre-training, or the common subject-specific fine-tuning. This may provide insight into better weight initialization, or integration of explicit early layers similar to Raghu et al. (2019) (one could also argue that SincNet layers Ravanelli and Bengio, 2018 are some such layers that could factor here). Additionally, as temporally minded reconstruction losses continue to develop (Rivest and Kohar, 2020), reconsidering the effectiveness of signal reconstruction as a pre-training objective (and/or regularization) is warranted, whether this is within an MLM-like scheme similar to BENDR, or a seq2seq model (Graves, 2012).

## 5. Conclusion

We have proposed MLM-like training as a self-supervised pre-training step for BCI/EEG DNNs. This is in the interest of diversifying the investigations into successful transfer learning schemes for DNNs applied to BCI and EEG with possible applicability to neuroimaging more generally. While previous approaches fashioned DNN transfer learning after ImageNet pre-training, we find this approach inadequate as there is limited applicable data availability and it is questionably analogous to its forebear. While our proposed alternative might similarly suffer from this latter point to some degree (the most distinct MLM success is with discrete sequences, not continuous ones), it is more conducive to leveraging potentially immense amounts of unlabeled data, it is not limited to long-term feature developments as with previous proposals, and it seems to produce representations equally suited to different users and sessions, which is a problem previous work appears less suited to solving. In summary, we see strong paths for the effective deployment of powerful computation and massive data scales with EEG and BCI. Effective solutions in these specific applications could help drive application *and* analysis solutions in neuroimaging and perhaps physiological signal analysis generally.

## Data Availability Statement

The original contributions presented in the study are included in the article/supplementary material, further inquiries can be directed to the corresponding author/s.

## Author Contributions

DK conceived of the presented idea, designed experiments, performed the analysis, drafted the manuscript, and designed the figures. DK developed implementation with assistance from SA-O. SA-O and FR edited manuscript. FR provided supervision throughout. All authors contributed to the article and approved the submitted version.

## Conflict of Interest

The authors declare that the research was conducted in the absence of any commercial or financial relationships that could be construed as a potential conflict of interest.

## Footnotes

^1^Consider the difficulty of collecting and labeling 100 more BCI trials as compared to the same for 100 more images.

^2^Though we believe that it is likely that a similar tendency to what we characterize herein holds for most applications of DNNs outside of their core artificial intelligence applications.

^3^While this is typical, some procedures, like covariance-based Riemannian classification schemes, do not necessarily need different features for different tasks (Lotte et al., 2018; Zanini et al., 2018). These are a very interesting exception to the argument we develop.

^4^With a strong latent representation, a nearest-neighbors labeling is sufficient to be nearly 100% accurate for some datasets, despite being recordings made on different hardware (Kostas and Rudzicz, 2020a).

^5^image-net.org/challenges/LSVRC/2012/

^6^https://www.image-net.org/index.php

^7^It is also worth noting that our own prior work does not consider or identify this.

^8^Terminology here can be somewhat fuzzy. What is meant by self-supervision is a supervision-like task that requires domain-relevant understanding in some sense. Sometimes, “semi-supervised” is used instead, as it is often also a semi-supervised procedure (Chen et al., 2016), since the task is learned in an unsupervised fashion first and then classic supervised learning is used with labels. Typically, though, semi-supervision involves inferring labels for unlabeled data during training. Instead, self-supervision is loosely a particular case of representation learning, which is not historically uncommon in BCI (Zhang et al., 2020b). Though this work is different given that typically the loss is domain or data agnostic.

^9^As is perhaps obvious in the name, though potentially misleading. Fine-tuning is the process of further training on a reserved portion of a target dataset, unless stated otherwise, this is typically through standard supervised training.

^10^https://github.com/SPOClab-ca/dn3

^11^https://www.kaggle.com/c/inria-bci-challenge

^12^The selection of four here was arbitrary.

^13^http://massdb.herokuapp.com/en/

## APPENDIX

### Downstream hyperparameters

**Table A1:** Hyperparameters that varied between datasets, and these were not changed between different model configurations (see list in section 2.4.2).

**Dataset**	**Batch Size**	**Epochs**	**Learning Rate**
MMI	4	7	1 × 10^−5^
BCIC	60	15	5 × 10^−5^
ERN	32	15	1 × 10^−5^
P300	80	20	1 × 10^−5^
SSC	64	40	5 × 10^−5^
